# Herpesvirus Entry Mediator Binding Partners Mediate Immunopathogenesis of Ocular Herpes Simplex Virus 1 Infection

**DOI:** 10.1128/mBio.00790-20

**Published:** 2020-05-12

**Authors:** Seo J. Park, Rachel E. Riccio, Sarah J. Kopp, Igal Ifergan, Stephen D. Miller, Richard Longnecker

**Affiliations:** a Department of Microbiology and Immunology, Northwestern University Feinberg School of Medicine, Chicago, Illinois, USA; Princeton University

**Keywords:** BTLA, CD160, HSV, HVEM, immunopathogenesis, herpesvirus stromal keratitis, LIGHT

## Abstract

Ocular herpes simplex virus 1 (HSV-1) infection leads to an immunopathogenic disease called herpes stromal keratitis (HSK), in which CD4^+^ T cell-driven inflammation contributes to irreversible damage to the cornea. Herpesvirus entry mediator (HVEM) is an immune modulator that activates stimulatory and inhibitory cosignals by interacting with its binding partners, LIGHT (TNFSF14), BTLA (B and T lymphocyte attenuator), and CD160. We have previously shown that HVEM exacerbates HSK pathogenesis, but the involvement of its binding partners and its connection to the pathogenic T cell response have not been elucidated. In this study, we investigated the role of HVEM and its binding partners in mediating the T cell response using a murine model of ocular HSV-1 infection. By infecting mice lacking the binding partners, we demonstrated that multiple HVEM binding partners were required for HSK pathogenesis. Surprisingly, while LIGHT^−/−^, BTLA^−/−^, and CD160^−/−^ mice did not show differences in disease compared to wild-type mice, BTLA^−/−^ LIGHT^−/−^ and CD160^−/−^ LIGHT^−/−^ double knockout mice showed attenuated disease characterized by decreased clinical symptoms, increased retention of corneal sensitivity, and decreased infiltrating leukocytes in the cornea. We determined that the attenuation of disease in HVEM^−/−^, BTLA^−/−^ LIGHT^−/−^, and CD160^−/−^ LIGHT^−/−^ mice correlated with a decrease in gamma interferon (IFN-γ)-producing CD4^+^ T cells. Together, these results suggest that HVEM cosignaling through multiple binding partners induces a pathogenic Th1 response to promote HSK. This report provides new insight into the mechanism of HVEM in HSK pathogenesis and highlights the complexity of HVEM signaling in modulating the immune response following ocular HSV-1 infection.

## Abstract (precis)

Herpes simplex virus 1 (HSV-1), a ubiquitous human pathogen, is capable of causing a progressive inflammatory ocular disease called herpes stromal keratitis (HSK). HSV-1 ocular infection leads to persistent inflammation in the cornea resulting in outcomes ranging from significant visual impairment to complete blindness. Our previous work showed that herpesvirus entry mediator (HVEM) promotes the symptoms of HSK independently of viral entry and that HVEM expression on CD45^+^ cells correlates with increased infiltration of leukocytes into the cornea during the chronic inflammatory phase of the disease. Here, we elucidated the role of HVEM in the pathogenic Th1 response following ocular HSV-1 infection and the contribution of HVEM binding partners in HSK pathogenesis. Investigating the molecular mechanisms of HVEM in promoting corneal inflammation following HSV-1 infection improves our understanding of potential therapeutic targets for HSK.

## INTRODUCTION

Ocular herpes simplex virus 1 (HSV-1) infection causes a destructive chronic inflammatory disease called herpes stromal keratitis (HSK) (1). HSK is characterized by inflammation of the corneal stroma, which promotes progressive corneal lesions and opacification, leading to severe visual impairment or blindness (2). Viral replication is required for the pathogenesis of HSK, but the corneal damage predominantly results from the invasion of polymorphonuclear neutrophils (PMN) and CD4^+^ T cells (3, 4). In a murine model of HSK, replicating HSV-1 is detected in the tear film from 0 to 6 days postinfection (dpi) and is primarily cleared by the innate immune response (3, 5). However, symptoms of ocular disease begin around 6 to 7 dpi and increase through day 14, coinciding with infiltration of PMNs and CD4^+^ T cells, which persist long after the replicating virus is no longer detectable (6, 7). Interferon gamma (IFN-γ)-producing and interleukin-17 (IL-17)-producing CD4^+^ T cells, in particular, have been implicated in driving infiltration of leukocytes to the cornea, causing increased severity of ocular disease (8–11). Previous studies highlighted the importance of pathogenic T cells in exacerbating HSK, but the molecular mechanisms mediating this inflammatory response have yet to be fully understood.

Herpesvirus entry mediator (HVEM) is a tumor necrosis factor (TNF) receptor superfamily member originally discovered as one of several entry receptors for HSV (12, 13). Interestingly, our recent *in vivo* studies revealed that the entry receptor function of HVEM is dispensable for the resulting pathogenesis observed following ocular HSV-1 infection (14). Ocular infections using HVEM^−/−^ mice show a significant reduction in clinical symptoms and infiltrating immune cells in the cornea, prompting an investigation of an alternative role of HVEM in ocular HSV-1 infection (7, 15). HVEM was previously found to modulate T cell-mediated immune responses by providing costimulatory and coinhibitory signaling through interaction with its binding partners, LIGHT (TNFSF14), BTLA (B and T lymphocyte attenuator), and CD160 (16) (see Fig. 6B). It exhibits bidirectional functionality by acting as either a receptor or a ligand, depending on the cellular and pathogenic conditions. HVEM, as a receptor, delivers costimulatory signals following interaction with its ligands, LIGHT, BTLA, and CD160, while HVEM, as a ligand, delivers coinhibitory signals through its receptors, BTLA and CD160 (17). Activation of HVEM signaling by LIGHT, BTLA, and CD160 promotes T cell activation, proliferation, cytokine production, and survival (18–20). BTLA and CD160 signaling activated by HVEM leads to repression of T cell responses (21, 22). Additionally, HVEM cosignaling is differentially regulated by its binding partners (17). For instance, a previous study revealed that the progression of experimental autoimmune uveitis was regulated by both HVEM-LIGHT and HVEM-BTLA interactions for HVEM-mediated stimulatory signaling, while another study described a protective role of HVEM-BTLA interactions in suppressing T cell-mediated responses to reduce the severity of experimental autoimmune encephalomyelitis (20–22). Our recent findings have shown that the absence of HVEM expression on CD45^+^ cells ameliorates corneal inflammation by causing a reduction in leukocyte infiltration, suggesting a role of HVEM in promoting the inflammatory immune response following ocular infection (7). However, the involvement of HVEM binding partners in regulating HSK pathogenesis has yet to be explored.

In this study, we investigated the contribution of HVEM binding partners in a murine model of ocular HSV-1 infection. We observed attenuated ocular pathology in BTLA^−/−^ LIGHT^−/−^ and CD160^−/−^ LIGHT^−/−^ double knockout mice but no differences between BTLA^−/−^ CD160^−/−^ mice and wild-type (WT) mice, indicating that multiple binding partners have differential roles in promoting disease. This attenuation in clinical symptoms in BTLA^−/−^ LIGHT^−/−^ and CD160^−/−^ LIGHT^−/−^ mice corresponded to a decreased number of infiltrating leukocytes in the corneas. Furthermore, we found reduced CD4^+^ T cell activation and production of IFN-γ in HVEM^−/−^ mice as well as BTLA^−/−^ LIGHT^−/−^ and LIGHT^−/−^ CD160^−/−^ mice, suggesting that HVEM has a stimulatory effect on the T cell response through interactions with multiple binding partners. Additionally, ocular HSV-1 infection of CD160^−/−^ LIGHT^−/−^ mice following treatment with an anti-BTLA monoclonal antibody (MAb) to fully abrogate HVEM signaling through its binding partners resulted in significant reductions in clinical symptoms and infiltrating leukocytes in the cornea as observed in HVEM^−/−^ mice. This report proposes a mechanism by which HVEM interaction with multiple binding partners exacerbates HSK through the induction of pathogenic IFN-γ-producing CD4^+^ T cells to drive chronic inflammation in the cornea.

## RESULTS

### HVEM binding partners alter ocular pathogenesis during the chronic inflammatory phase of HSK.

**FIG 1:**
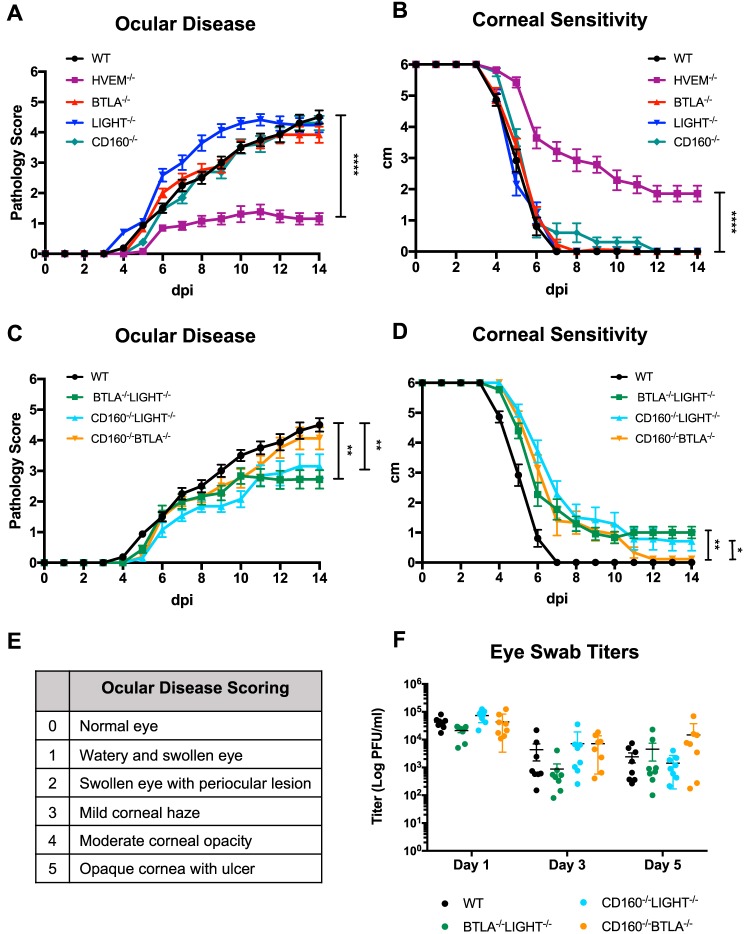
HVEM binding partners promote ocular disease following HSV-1 infection. C57BL/6 mice (8 to 10 weeks of age) were infected with 2.0 × 10^6^ PFU/5 μl per eye of HSV-1/17. (A) Ocular pathologies of WT, HVEM^−/−^, BTLA^−/−^, LIGHT^−/−^, and CD160^−/−^ mice were assessed daily using a scoring system from 0 to 5, with 5 being the most severe ocular pathology (*n* = 5 to 7 per group, 3 replicates). HVEM^−/−^ mice showed a significant difference in pathology score compared to WT mice 7 to 14 dpi (*P* < 0.0001). (B) Corneal touch sensitivities of WT, HVEM^−/−^, BTLA^−/−^, LIGHT^−/−^, and CD160^−/−^ mice were determined daily using a Leneau Cochet-Bonnet esthesiometer. Absence of a blink response at 0.5 cm was recorded as 0 cm (*n* = 5 to 7 per group, 3 replicates). HVEM^−/−^ mice showed a significant difference in blink response 6 to 14 dpi (*P* < 0.0001). (C) Ocular pathologies of BTLA^−/−^ LIGHT^−/−^, CD160^−/−^ LIGHT^−/−^, and CD160^−/−^ BTLA^−/−^ mice were assessed daily (*n* = 5 to 7 per group, 3 replicates). BTLA^−/−^ LIGHT^−/−^ mice showed a significant difference in ocular pathology compared to WT mice 11 to 14 dpi (*P* < 0.01), and CD160^−/−^ LIGHT^−/−^ mice showed a significant difference 10 to 14 dpi (*P* < 0.01). (D) Corneal touch sensitivities of BTLA^−/−^ LIGHT^−/−^, CD160^−/−^ LIGHT^−/−^, and CD160^−/−^ BTLA^−/−^ mice were determined daily (*n* = 5 to 7 per group, 3 replicates). BTLA^−/−^ LIGHT^−/−^ and CD160^−/−^ LIGHT^−/−^ mice showed a significant difference compared to WT mice 6 to 14 dpi (*P* < 0.05). Values from the experiments described in the panel A to panel D legends are expressed as means ± standard errors of the means (SEM) of results of analyses performed using two-way ANOVA with Holm-Sidak’s adjustment for multiple comparisons. (E) Table of ocular disease scoring used to determine disease severity as described in the panel A and panel C legends. (F) Viral titers in eye swabs of WT, BTLA^−/−^ LIGHT^−/−^, CD160^−/−^ LIGHT^−/−^, and CD160^−/−^ BTLA^−/−^ mice collected at days 1, 3, and 5 postinfection (*n* = 8 corneas). Values are expressed as means ± SEM of results of analyses performed using one-way ANOVA with Holm-Sidak’s adjustment for multiple comparisons (*, *P* < 0.05; **, *P* < 0.01; ***, *P* < 0.001; ****, *P* < 0.0001).

Our previous studies revealed that HVEM promotes ocular disease following HSV-1 infection through modulation of the immune response rather than through its role as a viral entry receptor (14). Because HVEM binding partners BTLA, LIGHT, and CD160 are known regulators of the immune modulatory function of HVEM, we investigated their contribution in altering ocular HSV-1 pathogenesis by infecting BTLA^−/−^, LIGHT^−/−^, and CD160^−/−^ mice. These mice were infected with 2.0 × 10^6^ PFU/eye of HSV-1 strain 17, and clinical symptoms of ocular disease were assessed daily with an established scoring system (Fig. 1E). WT, BTLA^−/−^, LIGHT^−/−^, and CD160^−/−^ mice developed symptoms that appeared first around 6 dpi and continued to worsen over 14 days, while HVEM^−/−^ mice exhibited significantly attenuated symptoms that did not increase in severity (Fig. 1A). The levels of severity of disease symptoms in BTLA^−/−^, LIGHT^−/−^, and CD160^−/−^ mice were comparable to those seen in WT mice, as indicated by the clinical scores. We previously reported that the severity of clinical symptoms directly correlates with loss of corneal sensitivity (7). To further assess the contribution of HVEM binding partners in disease severity, we used a Luneau Cochet-Bonnet esthesiometer to measure the blink response in BTLA^−/−^, LIGHT^−/−^, and CD160^−/−^ mice following HSV-1 ocular infection. We observed a complete loss of corneal sensitivity in these mice by 14 dpi, which was similar to the results seen with WT mice, while the HVEM^−/−^ mice retained the blink response (Fig. 1B). These results suggest that the presence of single HVEM binding partners is insufficient to alter clinical symptoms of ocular HSV-1 infection. However, because of the differential roles of HVEM binding partners in various disease models previously described by other groups, we cannot rule out the possibility of the roles of multiple binding partners in contributing to ocular disease pathogenesis. In particular, Sakoda et al. reported contributions by both BTLA and LIGHT in mediating the HVEM cosignals that stimulate the progression of experimental autoimmune uveitis (23).

Based on these earlier data and our work identifying HVEM as an immune modulator in our disease model, we investigated the possibility of the involvement of multiple binding partners in altering ocular disease following HSV-1 infection. We infected BTLA^−/−^ LIGHT^−/−^, CD160^−/−^ LIGHT^−/−^, and CD160^−/−^ BTLA^−/−^ double knockout mice and used the same methods to assess disease severity. BTLA^−/−^ LIGHT^−/−^ and CD160^−/−^ LIGHT^−/−^ mice showed significantly reduced clinical symptoms from 11 dpi to 14 dpi compared to WT mice (Fig. 1C). We also observed significant retention of corneal sensitivity in BTLA^−/−^ LIGHT^−/−^ and CD160^−/−^ LIGHT^−/−^ mice, in contrast to the WT mice, which had lost all blink responses by 7 dpi (Fig. 1D). CD160^−/−^ BTLA^−/−^ mice lost all blink responses by 12 dpi, which correlated with the more severe clinical symptoms observed in these mice than in the BTLA^−/−^ LIGHT^−/−^ and CD160^−/−^ LIGHT^−/−^ mice. Despite these differences in corneal sensitivity at 14 dpi, all of the double knockout mice showed similar delays in loss of corneal sensitivity compared to the WT mice and retained blink response through 10 dpi. These results indicate that multiple HVEM binding partners contribute simultaneously to HSV-1 ocular disease.

Interestingly, the CD160^−/−^ BTLA^−/−^ mice showed development and severity of disease similar to the results seen with WT mice, while the BTLA^−/−^ LIGHT^−/−^ and CD160^−/−^ LIGHT^−/−^ mice exhibited significantly reduced disease symptoms, suggesting the possibility that LIGHT is a major binding partner promoting ocular disease. Previous studies revealed that LIGHT acts as a major ligand of HVEM to activate downstream NF-κB signaling, which promotes proinflammatory signals in T cells. BTLA and CD160 can also act as ligands to stimulate HVEM signaling but were previously shown to have their own receptor activity to deliver downstream inhibitory signaling (18–20). This suggests that the ocular disease shown by CD160^−/−^ BTLA^−/−^ mice was similar to that seen with WT mice because LIGHT is available to deliver proinflammatory signaling through HVEM, without the presence of BTLA and CD160 inhibitory receptor activity. Additionally, BTLA^−/−^ LIGHT^−/−^ and CD160^−/−^ LIGHT^−/−^ mice showed a reduction in clinical symptoms and loss of corneal sensitivity compared to WT mice, but the levels did not match the significant attenuation of disease observed in HVEM^−/−^ mice. This indicates that because BTLA and CD160 are able to act as ligands to stimulate HVEM signaling, the absence of two of three binding partners is not sufficient to cause the reduction in disease pathogenesis observed in HVEM^−/−^ mice. Therefore, we predict that all three binding partners contribute to HSV-1 ocular disease.

To determine if the differences in ocular disease pathogenesis were a result of differences in viral replication early in infection, we measured viral titers in the tear film at 1, 3, and 5 dpi in BTLA^−/−^ LIGHT^−/−^, CD160^−/−^ LIGHT^−/−^, and CD160^−/−^ BTLA^−/−^ mice (Fig. 1F). There was no difference in viral titers between the WT mice and the double knockout mice, indicating that the observed clinical symptoms and corneal sensitivity were not due to early events that altered viral replication. These findings suggest that HVEM binding partners may promote ocular disease pathogenesis by altering the immune response through HVEM, further supporting our previous work investigating the viral entry receptor-independent, immune modulatory role of HVEM in HSK pathogenesis.

### HVEM binding partners promote leukocyte infiltration in the cornea.

**FIG 2:**
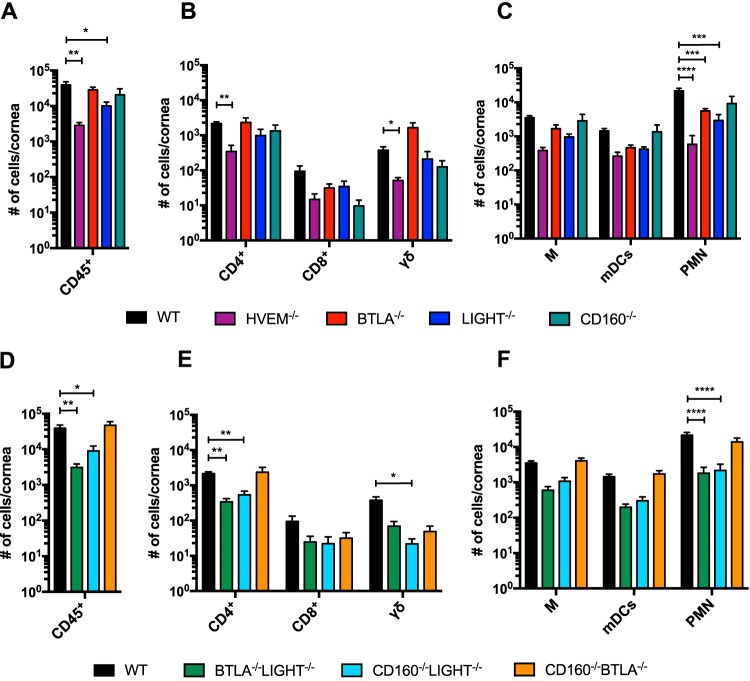
BTLA, CD160, and LIGHT differentially contribute to increased leukocytic infiltration in the cornea during the clinical phase of ocular infection. Leukocytic infiltrates isolated from corneas from mice infected with 2.0 × 10^6^ PFU/5 μl per eye of HSV-1/17 were analyzed by flow cytometry at 14 days postinfection (dpi). (A) Total numbers of leukocytes in WT, HVEM^−/−^, BTLA^−/−^, LIGHT^−/−^, and CD160^−/−^ mice were determined by CD45 staining (*n* = 5 to 7 per group, 3 replicates). (B) Cell surface staining for CD4^+^, CD8^+^, and γδ TCR within CD45^+^ and CD3^+^ populations was performed, and absolute cell counts of WT, HVEM^−/−^, BTLA^−/−^, LIGHT^−/−^, and CD160^−/−^ corneas were calculated from total numbers of cells (*n* = 5 to 7 per group, 3 replicates). (C) Within CD45^+^ and CD11b^+^ populations of cells from WT, HVEM^−/−^, BTLA^−/−^, LIGHT^−/−^, and CD160^−/−^ mice, identities of myeloid cell lineage cell compartments were determined using the following gating strategy: monocytes/macrophages (M; Ly6G^−^ CD11c^−^), myeloid dendritic cells (mDCs; Ly6G^−^ CD11c^+^), and polymorphonuclear neutrophils (PMN; Ly6G^+^ Ly6C^+^) (*n* = 5 to 7 per group, 3 replicates). (D) Total number of CD45^+^ cells in BTLA^−/−^ LIGHT^−/−^, CD160^−/−^ LIGHT^−/−^, and CD160^−/−^ BTLA^−/−^ mouse corneas (*n* = 5 to 7 per group, 3 replicates). (E) Lymphoid lineage cells from BTLA^−/−^ LIGHT^−/−^, CD160^−/−^ LIGHT^−/−^, and CD160^−/−^ BTLA^−/−^ mouse corneas (*n* = 5 to 7 per group, 3 replicates). (F) Myeloid cell lineages from BTLA^−/−^ LIGHT^−/−^, CD160^−/−^ LIGHT^−/−^, and CD160^−/−^ BTLA^−/−^ mouse corneas (*n* = 5 to 7 per group, 3 replicates). Values are expressed as means ± SEM analyzed using one-way ANOVA with Holm-Sidak’s adjustment for multiple comparisons (*, *P* < 0.05; **, *P* < 0.01; ***, *P* < 0.001; ****, *P* < 0.0001).

A hallmark of HSK pathogenesis is the infiltration of leukocytes late in infection that causes chronic inflammation and damage to the cornea (1). Leukocyte infiltration following ocular HSV-1 infection is biphasic; the first wave occurs 0 to 6 dpi, consisting of polymorphonuclear cells (PMNs), macrophages, natural killer (NK) cells, dendritic cells (DCs), and γδ T cells (3, 24–26). In the second wave, leukocytes invade the cornea from 8 to 20 dpi following viral clearance, causing corneal inflammation and clinical symptoms of HSK (27). We previously showed that at 14 dpi, corneas of HVEM^−/−^ mice contained a significantly lower number of infiltrating leukocytes than those of WT mice, indicating that HVEM promotes infiltration of immune cells into the cornea following ocular HSV-1 infection (7). We hypothesized that HVEM binding partners regulate corneal inflammation by serving as ligands for HVEM-mediated stimulatory signaling. To investigate the specific involvement of the HVEM binding partners in altering infiltrating leukocytes, BTLA^−/−^, LIGHT^−/−^, and CD160^−/−^ mice were infected, and their corneas were harvested and analyzed by flow cytometry at 14 dpi. We also performed the same experiment on WT and HVEM^−/−^ mice for a direct comparison with the binding partner knockout mice (Fig. 2A to C). Corneas from LIGHT^−/−^ mice contained significantly lower numbers of total CD45^+^ cells than those from WT mice, indicating that LIGHT may have a role in promoting leukocyte infiltration into the cornea (Fig. 2A). Upon analysis of leukocyte subtypes in the lymphoid and myeloid lineages, we observed that both BTLA^−/−^ and LIGHT^−/−^ mouse corneas contained lower number of PMNs, the cell type previously identified to cause corneal damage during HSK (Fig. 2C). CD160^−/−^ mice did not show a significant difference in infiltrating cells compared to WT, but this was not surprising as CD160 expression is restricted to NK cells, NKT cells, γδ T cells, and only a small percentage of CD4^+^ and CD8^+^ T cells (28–30).

We performed the same analysis with BTLA^−/−^ LIGHT^−/−^, CD160^−/−^ LIGHT^−/−^, and CD160^−/−^ BTLA^−/−^ double knockout mice and observed a significant reduction in infiltration of CD45^+^ cells in corneas of BTLA^−/−^ LIGHT^−/−^ and CD160^−/−^ LIGHT^−/−^ mice compared to WT mice whereas the numbers of CD45^+^ cells in CD160^−/−^ BTLA^−/−^ corneas were comparable to those detected in WT corneas (Fig. 2D). The reductions in infiltrating leukocytes in the BTLA^−/−^ LIGHT^−/−^ and CD160^−/−^ LIGHT^−/−^ mice indicate that multiple HVEM binding partners play roles in promoting leukocyte infiltration in the cornea following infection. However, the CD160^−/−^ BTLA^−/−^ mice did not show any reduction in infiltrating cells, indicating that the individual functions of the binding partners appear to be more complex than simply serving as ligands for HVEM-mediated stimulatory signaling. Identification of the cell types that infiltrated the cornea revealed that the BTLA^−/−^ LIGHT^−/−^ and CD160^−/−^ LIGHT^−/−^ mouse corneas specifically contained lower numbers of CD4^+^ T cells and PMNs (Fig. 2E and F). Additionally, the CD160^−/−^ LIGHT^−/−^ mice exhibited a decreased number of γδ T cells in the corneas comparable to the level seen with the HVEM^−/−^ mice (Fig. 2E). This is particularly important because CD4^+^ T cells, γδ T cells, and PMNs have a pathogenic role late in ocular HSV-1 infection. CD4^+^ and γδ T cells secrete proinflammatory cytokines, including IFN-γ and IL-17, and PMNs produce oxy-radicals and metalloproteinases which directly contribute to the development of immunopathological damage in the cornea (8, 10, 11, 31). The reduced number of PMNs and CD4^+^ T cells observed in BTLA^−/−^ LIGHT^−/−^ and CD160^−/−^ LIGHT^−/−^ mouse corneas may have been due to the loss of ligands to activate HVEM cosignaling.

### Activation and expansion of CD4^+^ T cells are impaired in the absence of HVEM and its binding partners.

**FIG 3:**
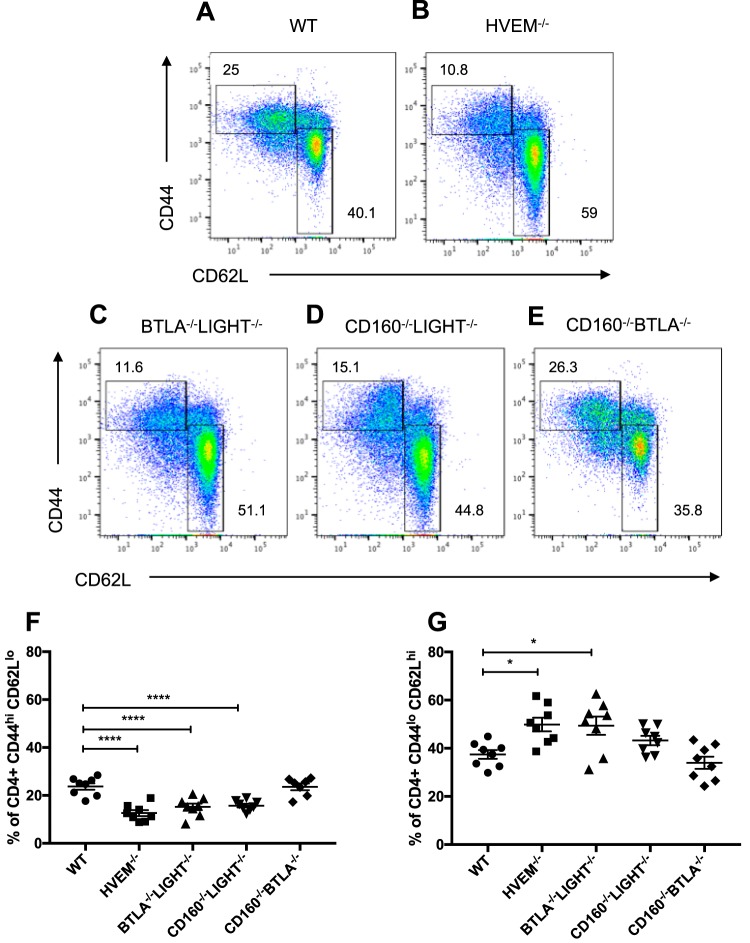
HVEM cosignaling with multiple binding partners results in increased activation and expansion of CD4^+^ T cells in DLNs during the clinical phase of ocular infection. Submandibular lymph node cells were isolated from mice infected with HSV-1 at 14 dpi, and T cell activation markers were analyzed by flow cytometry. (A to E) Representative FACS plots of the expression of CD44 and CD62L on live CD4^+^ cells within CD45^+^ and CD3^+^ populations. (F) Frequency of live effector CD4^+^ T cells (CD44^hi^ CD62L^lo^). (G) Frequency of live naive CD4^+^ T cells (CD44^lo^ CD62L^hi^). (*n* = 4, 2 replicates). Values shown in panels B and C are means ± SEM of results from analyses performed using ordinary one-way ANOVA (*, *P* < 0.05; **, *P* < 0.01; ***, *P* < 0.001; ****, *P* < 0.0001).

Past studies showed that CD4^+^ T cells cause damage to the cornea by producing proinflammatory cytokines and promoting infiltration of leukocytes to the cornea late in ocular infection (11, 32). Because HVEM mediates T cell cosignaling through the engagement of its binding partners, we hypothesized that HVEM and its binding partners alter activation and expansion of T cells, subsequently leading to the pathogenic T cell driven ocular inflammation. In order to investigate the effect of HVEM on the CD4^+^ T cell population, we infected WT and HVEM^−/−^ mice and isolated cells in the draining lymph nodes (DLNs) at 14 dpi for flow cytometry analysis. The frequency of CD44^hi^ CD62L^lo^ effector CD4^+^ T cells was significantly reduced in HVEM^−/−^ mice compared to WT mice, indicating decreased expansion of CD4^+^ T cells in the absence of HVEM (Fig. 3A and B). This result correlated with an increased frequency of CD44^lo^ CD62L^hi^ naive CD4^+^ T cells in HVEM^−/−^ mice (Fig. 3G). These data demonstrate that HVEM promotes T cell activation and expansion in the DLNs, which increases the number of T cells that can potentially migrate to the cornea to cause inflammation. Furthermore, this function of HVEM could explain the differences between WT and HVEM^−/−^ mice in the observed elevated levels of leukocyte infiltration in the cornea (Fig. 2).

To elucidate the contribution of HVEM binding partners in increasing activated T cells, we performed the same experiment using LIGHT^−/−^, BTLA^−/−^, and CD160^−/−^ mice and did not observe differences in the frequency of CD44^hi^ CD62L^lo^ effector CD4^+^ T cells in these DLNs compared to WT DLNs (data not shown). This was expected, as the data from the experiments whose results are shown in Fig. 1 and 2 indicated no significant differences in disease pathogenesis in these mice compared to WT mice. We predicted that the double knockout mice would exhibit differences in effector CD4^+^ T cells because of the observed reduction in infiltrating immune cells in the corneas (Fig. 2). BTLA^−/−^ LIGHT^−/−^, CD160^−/−^ LIGHT^−/−^, and CD160^−/−^ BTLA^−/−^ mice were infected, and the cells from DLNs were analyzed at 14 dpi. CD4^+^ T cells from BTLA^−/−^ LIGHT^−/−^ and CD160^−/−^ LIGHT^−/−^ mice exhibited reduced frequencies of CD44^hi^ CD62L^lo^ effector CD4^+^ T cells compared to those from WT mice, and this reduction corresponded to the higher frequency of CD44^lo^ CD62L^hi^ naive CD4^+^ T cells in these mice (Fig. 3C and D). BTLA^−/−^ LIGHT^−/−^ and CD160^−/−^ LIGHT^−/−^ mice showed similar levels of CD44^hi^ CD62L^lo^ effector CD4^+^ T cells compared to HVEM^−/−^ mice, indicating that HVEM alters T cell activation following ocular HSV-1 infection through cosignaling with its binding partners (Fig. 3F). However, CD160^−/−^ BTLA^−/−^ and WT mice showed comparable frequencies of CD4^+^CD44^hi^ CD62L^lo^ cells and CD4^+^CD44^lo^ CD62L^hi^ cells (Fig. 3E), suggesting that not all binding partners function as ligands for HVEM to promote T cell activation and expansion.

### HVEM and its binding partners promote IFN-γ-producing CD4^+^ T cells in the DLNs following infection.

**FIG 4:**
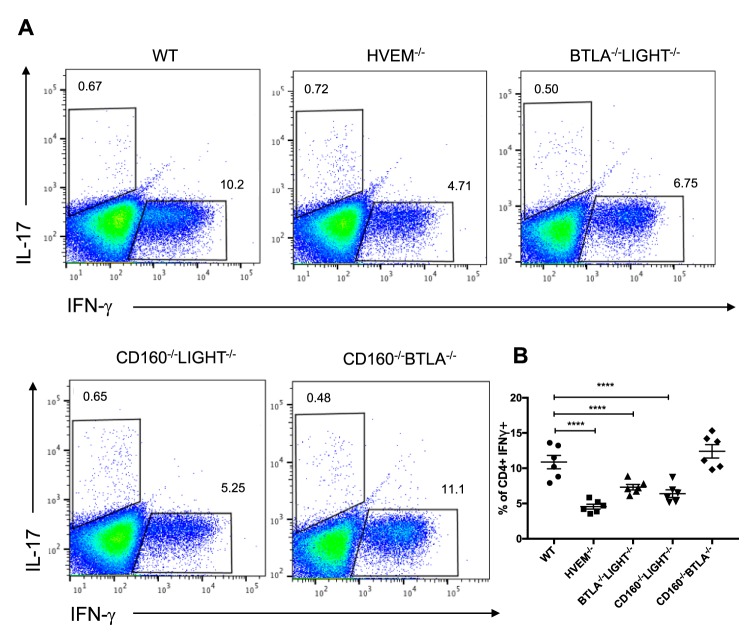
HVEM contributes to elevated levels of IFN-γ-expressing CD4^+^ T cells in the DLNs following HSV-1 infection through its cosignaling with its binding partners. Cells from submandibular lymph nodes were isolated at 14 dpi and stimulated with PMA/ionomycin to measure intracellular cytokine secretion levels. (A) Representative FACS plots of IFN-γ-producing live CD4^+^ T cells gated on CD45^+^ and CD4^+^. (B) Percentages of IFN-γ-producing live CD4^+^ T cells (*n* = 3, 2 replicates). Values are means ± SEM of results of analyses performed using ordinary one-way ANOVA (*, *P* < 0.05; **, *P* < 0.01; ***, *P* < 0.001; ****, *P* < 0.0001).

IFN-γ plays a pathological role during the late stage of ocular HSV-1 infection by activating the neutrophils that cause direct damage to the cornea and stimulating the secretion of other proinflammatory cytokines (11, 33). HVEM cosignaling was found to promote Th1 responses in various disease models (34–36). Therefore, we hypothesized that HVEM contributes to chronic inflammation during ocular HSV-1 infection by promoting the Th1 response following infection. Ocular infection of WT and HVEM mice was performed, and DLNs were harvested at 14 dpi. Stimulation of DLN cells with phorbol 12-myristate 13-acetate 40 (PMA)/ionomycin revealed that the frequency of WT CD4^+^ T cells that produced IFN-γ was higher than that of HVEM^−/−^ CD4^+^ T cells (Fig. 4). To assess the contribution of HVEM binding partners in altering the Th1 response, the same analysis was performed in LIGHT^−/−^, BTLA^−/−^, and CD160^−/−^ mice, and no differences in the frequencies of Th1 cells were observed (data not shown). However, when BTLA^−/−^ LIGHT^−/−^, CD160^−/−^ LIGHT^−/−^, and CD160^−/−^ BTLA^−/−^ mice were infected, the BTLA^−/−^ LIGHT^−/−^ and CD160^−/−^ LIGHT^−/−^ DLNs contained a significantly lower frequency of CD4^+^ T cells that produced IFN-γ than the WT DLNs, while there was no significant difference between the CD160^−/−^ BTLA^−/−^ and WT DLNs in the frequencies of those cells (Fig. 4B). The decreased levels of IFN-γ-producing CD4^+^ T cells in HVEM^−/−^ mice provide evidence indicating that HVEM promotes the Th1 response that exacerbates HSK pathogenesis. The frequencies of IFN-γ-producing CD4^+^ T cells in BTLA^−/−^ LIGHT^−/−^ and CD160^−/−^ LIGHT^−/−^ mice were comparable to those in HVEM^−/−^ mice, suggesting an involvement of HVEM cosignaling through its binding partners.

### Anti-BTLA MAb treatment of CD160^−/−^ LIGHT^−/−^ mice reduces ocular pathogenesis and infiltration of leukocytes in the cornea.

**FIG 5:**
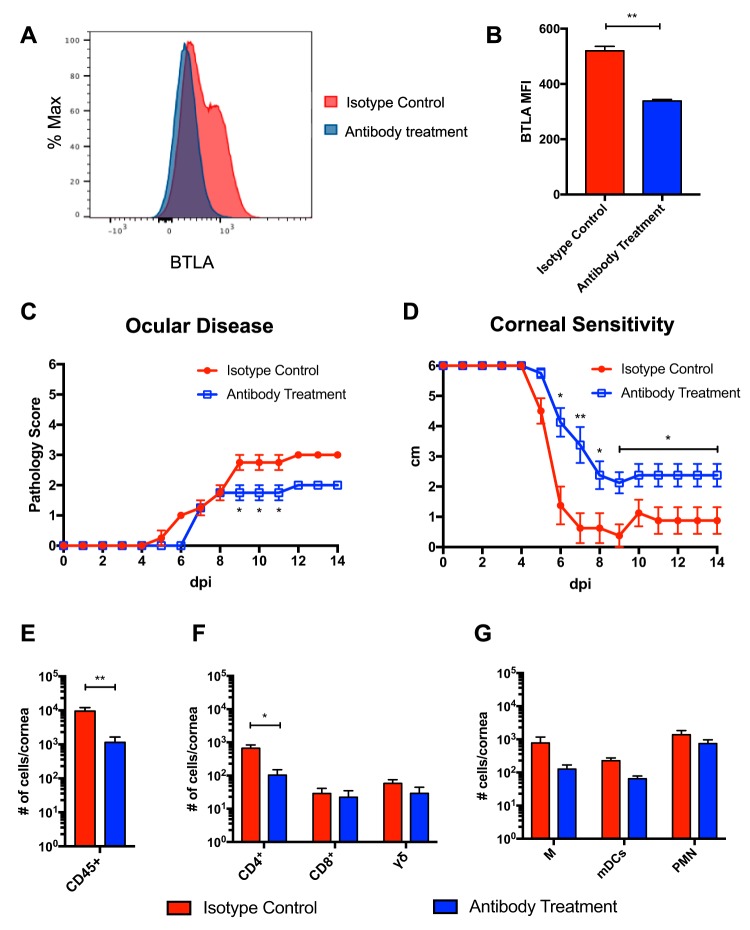
Treatment of CD160^−/−^ LIGHT^−/−^ mice with the anti-BTLA MAb PJ196 reduces severity of ocular pathology and decreases leukocyte infiltration in the cornea. CD160^−/−^ LIGHT^−/−^ mice (8 to 10 weeks of age) were treated with 200 μg of PJ196 MAb or isotype control Ab intraperitoneally. (A) Splenocytes were isolated from Ab-treated mice at 14 dpi and stained for BTLA expression. (B) Mean fluorescence intensity of BTLA on CD45^+^ cells. (C) Ocular pathology was assessed daily using a scoring system. (D) Corneal touch sensitivity was measured daily using a Leneau Cochet-Bonnet esthesiometer. (E) Total numbers of infiltrating leukocytes in the cornea 14 dpi were determined by CD45^+^ staining. (F and G) Total numbers of lymphoid cells (F) and myeloid cells (G) were determined by flow cytometry using the markers and gating strategy described for Fig. 2 (*n* = 4, 2 replicates). Values in panels C to G are means ± SEM of results of analyses performed with two-tailed *t* test with Holm-Sidak’s correction for multiple comparisons (*, *P* < 0.05; **, *P* < 0.01; ***, *P* < 0.001; ****, *P* < 0.0001).

BTLA^−/−^ LIGHT^−/−^ and CD160^−/−^ LIGHT^−/−^ mice showed decreased levels of ocular pathology and infiltrating immune cells in the cornea compared to WT mice. However, the HVEM^−/−^ mice exhibited an even greater reduction in disease pathology and infiltrating immune cells, suggesting that all three binding partners may play a role in driving ocular pathogenesis. In order to investigate the contribution of all three binding partners, CD160^−/−^ LIGHT^−/−^ mice were treated with anti-BTLA MAb PJ196, a nondepleting antibody that downregulates BTLA surface expression (37). Splenocytes from mice treated with PJ196 or isotype control confirmed a decreased number of BTLA^+^ CD45^+^ cells from the PJ196 MAb-treated mice (Fig. 5A). The mean fluorescence intensity (MFI) indicated that the surface expression of BTLA was downregulated in CD45^+^ cells (Fig. 5B). CD160^−/−^ LIGHT^−/−^ mice that received PJ196 showed decreased ocular pathology (Fig. 5C) and improved corneal sensitivity (Fig. 5D) compared to the CD160^−/−^ LIGHT^−/−^ mice that received the isotype control. Analysis of the infiltrating leukocytes in the cornea by flow cytometry at 14 dpi revealed that MAb treatment significantly reduced the levels of CD45^+^ cells (Fig. 5E), specifically those of CD4^+^ T cells (Fig. 5F). Levels of myeloid cells were also reduced in the MAb treatment group compared to the isotype control group, but this result did not reach statistical significance (Fig. 5G). These findings indicate that the cosignaling between HVEM and its binding partners LIGHT, BTLA, and CD160 plays a significant role in inducing the inflammatory response during HSK.

## DISCUSSION

In this study, we investigated the role of HVEM and its binding partners in promoting the pathogenic T cell response during the chronic inflammatory phase of herpes stromal keratitis. Our data indicate that HVEM contributes to HSK immunopathology by upregulating activation and expansion of the CD4^+^ T cells that secrete IFN-γ following infection. the presence of BTLA, LIGHT, and CD160, known binding partners of HVEM, was found to increase the inflammatory response during the chronic phase of HSK. Additionally, we demonstrated that treatment with an anti-BTLA MAb in LIGHT^−/−^ CD160^−/−^-infected mice, which downregulates BTLA expression, reduced levels of ocular disease, indicating that HVEM stimulatory cosignaling through these binding partners is a major contributor of HSK pathogenesis.

Our previous findings showed that HVEM exacerbates HSK pathology independently of its role as an entry receptor (7, 14). We further demonstrated that HVEM expressed on CD45^+^ cells limited infiltration of leukocytes into the cornea, minimizing the damage caused by CD4^+^ T cells and PMNs (7). It has long been established that T cell-deficient mice fail to develop HSK but that the transfer of exogenous CD4^+^ T cells restores ocular disease (4, 38). Depletion of T cells using antibodies also reduces symptoms of HSK (39), indicating that T cells are major drivers of HSK pathogenesis. Because CD4^+^ T cells are the primary orchestrators of HSK pathogenesis that facilitate the infiltration of PMNs and other immune cells during the chronic inflammatory phase of HSK, we chose to characterize the role of HVEM in altering the T cell response. HVEM and its binding partners have been shown to play a crucial role in inflammatory diseases by mediating stimulatory and inhibitory cosignals to T cells (17). These cosignals modulate T cell responses by influencing their activation, differentiation, and survival. Previous reports demonstrated the pathogenic function of Th1 cells in HSK pathology (4, 10). Th1 cells were found to be the major CD4^+^ T cell type that infiltrates the cornea following HSV-1 infection, and IFN-γ secretion by these cells promotes the inflammatory milieu in the cornea. Furthermore, IFN-γ stimulates cytokine secretion and PMN activation, and it was previously reported that anti-IFN-γ treatment during the chronic inflammatory phase of HSK following viral clearance reduced inflammation and damage to the cornea during ocular HSV-1 infection (11). We found that HVEM promoted activation and expansion of effector T cells following ocular HSV-1 infection and increased induction of the Th1-type T cell response. These results could be explained by the known receptor activity of HVEM, which delivers downstream costimulatory signals to cells by inducing NF-κB activation (40–42). Th17 cells have also been shown to contribute to HSK pathogenesis, but our studies indicated that HVEM altered IFN-γ production but not IL-17 production in CD4^+^ T cells at the time points investigated. These data provide an explanation for how HVEM exacerbates HSK pathology. We previously observed that HVEM expression correlated with increased leukocytic infiltration and corneal inflammation, and in this study we found that HVEM has a role in elevating the pathogenic Th1 response, a known major driver of immune cell infiltration in the cornea during HSK.

We further characterized the possible molecular mechanisms of HVEM in promoting HSK pathogenesis by investigating the contribution of its binding partners during disease. LIGHT, BTLA, and CD160 are cognate binding partners of HVEM that modulate immune responses (16, 17). LIGHT (TNFSF14) in particular has been found to deliver costimulatory signals through HVEM, resulting in enhancement of proliferation and cytokine secretion in T cells (43). Transgenic mice with constitutive expression of LIGHT on T cells developed spontaneous autoimmune disorders due to the overactivation of T cells (44). From these results, we hypothesized that LIGHT-HVEM signaling plays a role in exacerbating HSK pathogenesis, and we expected LIGHT^−/−^ mice to behave similarly to HVEM^−/−^ mice following infection. However, we did not observe differences in ocular pathology and loss of corneal sensitivity in LIGHT^−/−^ mice following HSV-1 infection compared to WT mice, although there was some reduction in the level of PMN infiltration of the LIGHT^−/−^ cornea. Because we did not observe significant differences in ocular disease, we could not conclude that the evidence showing a reduction in the numbers of PMNs was sufficient for us to conclude that LIGHT-HVEM signaling alone was a major player in driving HSK pathogenesis. We next investigated the possible contribution of two other HVEM binding partners, BTLA and CD160, which could explain why LIGHT^−/−^ mice showed similar levels of disease pathogenesis comparable to that seen with WT mice. BTLA and CD160 downstream signaling inhibits T cell activation during expression on T cells (28, 45). Previous reports indicated that the absence of BTLA resulted in increased susceptibility to autoimmune disorders, providing evidence of the inhibitory function of BTLA (45, 46). However, both BTLA and CD160 can also act as ligands for HVEM to deliver a stimulatory signal to T cells (19). We explored the possibility that BTLA and CD160 could play a role in the chronic inflammation observed in HSK. Surprisingly, the results that we observed in BTLA^−/−^ and CD160^−/−^ mice were similar to those that we observed in LIGHT^−/−^ mice. BTLA^−/−^ corneas also showed a reduction in infiltrating PMNs, indicating the possibility that BTLA acts as a ligand, similarly to LIGHT, to deliver stimulatory signals through HVEM. These data suggested that multiple binding partners could be acting as ligands for HVEM to exacerbate ocular disease pathogenesis.

Upon observation of BTLA^−/−^ LIGHT^−/−^, CD160^−/−^ LIGHT^−/−^, and CD160^−/−^ BTLA^−/−^ mice following ocular HSV-1 infection, we were able to determine that HVEM downstream signaling is the major pathway involved in exacerbating HSK pathogenesis and that all three binding partners act as functional ligands for HVEM. However, we observed that each binding partner also had some role as a receptor in altering ocular pathology and immune response during HSK. The BTLA^−/−^ LIGHT^−/−^ and CD160^−/−^ LIGHT^−/−^ mice showed a reduction in ocular pathology and improved retention of corneal sensitivity. This could have been due to the absence of ligands delivering stimulatory signals through HVEM-expressing T cells, but we cannot rule out the involvement of CD160 in BTLA^−/−^ LIGHT^−/−^ mice and BTLA in CD160^−/−^ LIGHT^−/−^ mice in delivering inhibitory downstream signals to dampen the T cell response. Interestingly, CD160^−/−^ BTLA^−/−^ mice showed a WT-like phenotype, indicating that in the absence of BTLA and CD160, the costimulatory ligand, LIGHT, is the major player in inducing corneal inflammation and subsequent disease pathology in HSK. We further investigated the hypothesis that HVEM downstream cosignaling is the major pathway in HSK pathogenesis by treating CD160^−/−^ LIGHT^−/−^ mice with PJ196, the anti-BTLA MAb that specifically downregulates surface expression of BTLA without depleting BTLA^+^ cells. This antibody was used to investigate HSK pathogenesis in the absence of all three HVEM binding partners. The MAb treatment produced a result similar to a disease phenotype seen in HVEM^−/−^ mice, indicating that HVEM downstream costimulatory signaling plays a major role in HSK pathogenesis.

**FIG 6:**
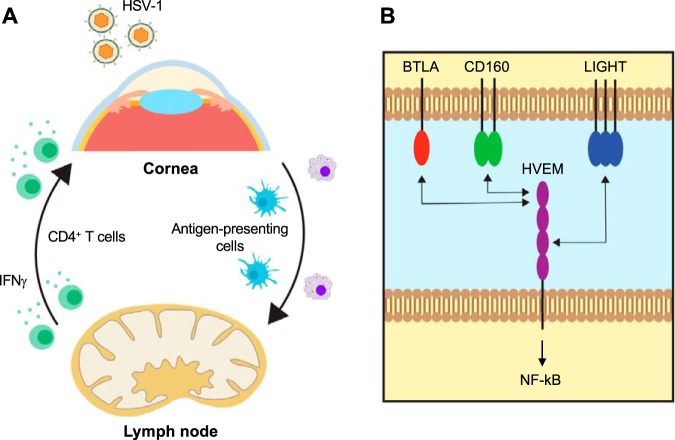
The role of HVEM binding partners in HSV-1 infection. (A) Proposed model of the immune response following ocular HSV-1 infection. HSV-1 infection of the cornea leads to the migration of antigen-presenting cells to the draining lymph nodes (DLN). HVEM cosignaling through its binding partners could function in the cornea or the DLNs to stimulate activation of immune cells. Antigen-presenting cells in the DLNs induce activation of expansion of IFN-γ-producing CD4^+^ Th1 cells, which can migrate to the cornea to promote infiltration of immune cells resulting in the corneal damage observed in HSK. (B) HVEM binding partner network. We hypothesize that HVEM interacts with multiple binding partners in both the cornea and the DLNs to promote an aberrant immune response triggered by HSV-1 infection. These interactions activate NF-κB signaling, promoting proliferation and survival of CD4^+^ T cells, which contribute to the exacerbation of HSK.

Our proposed model of the mechanism of HVEM in HSK illustrates the complexity of HVEM signaling (Fig. 6A). Following corneal HSV-1 infection, antigen-presenting cells travel to the DLNs, resulting in the activation and expansion of IFN-γ-producing CD4^+^ T cells. These effector T cells infiltrate the cornea and produce IFN-γ, which upregulates chemokine ligand expression to attract more immune cells to the cornea, causing inflammation and subsequent symptoms of HSK (Fig. 6A). HVEM signaling modulates the T cell response summarized in Fig. 6A by interacting with its binding partners in the cornea and the DLNs (Fig. 6B). Our findings suggest that HVEM activates its known downstream NF-κB signaling through multiple binding partner interactions and that NF-κB signaling stimulates proliferation and survival of T cells to exacerbate HSK.

The differential involvement of HVEM binding partners that we discovered may be due to differences in their spatiotemporal expression on immune cells. LIGHT expression is restricted to immature dendritic cells and activated T cells, while BTLA is widely expressed in T cells, B cells, DCs, macrophages, and NK cells (34, 47–50). CD160 is expressed in NK cells, NKT cells, γδ T cells, and a small subset of CD4^+^ and CD8^+^ T cells (28, 30). Therefore, the three binding partners are important in stimulating HVEM cosignaling in different immune cell populations during HSK pathogenesis. Additionally, multiple binding partners can interact simultaneously with HVEM and these interactions can occur in *cis* or *trans*, thereby complicating the analysis of the individual role of each receptor (19, 51–53).

Although further investigation is needed to identify the functional differences between the downstream pathways of HVEM and each of the binding partners, we have revealed that HVEM receptor cosignaling triggered by its binding partners is a major pathway stimulating the pathogenic T cell response that exacerbates HSK pathogenesis. Our findings highlight a level of complexity of HVEM signaling that may complicate the development of targeted therapies for HSK, necessitating therapies that act on multiple components of the immune response.

## MATERIALS AND METHODS

### Ethics statement.

This study was carried out in adherence to the recommendations in the Guide for the Care and Use of Laboratory Animals of the National Institutes of Health. The protocol was approved by the Institutional Animal Care and Use Committee (IACUC) of Northwestern University. Procedures were performed with ketamine/xylazine or isoflurane anesthesia to minimize suffering.

### Cells and virus.

HSV-1 strain 17 was obtained from David Leib (Dartmouth Medical School, Hanover, NH) and propagated in Vero cells and stored at −80°C until use. Plaque assay was used to determine viral titers of eye swabs as described previously (14).

### Mice.

C57BL/6 mice were purchased from Jackson Laboratory (Bar Harbor, ME). The origins of the HVEM^−/−^, LIGHT^−/−^, BTLA^−/−^, and CD160^−/−^ mice used in the studies have been described previously (30, 49, 54). LIGHT^−/−^ BTLA^−/−^, BTLA^−/−^ CD160^−/−^, and CD160^−/−^ LIGHT^−/−^ mice were generated by crossing LIGHT^−/−^, BTLA^−/−^, and CD160^−/−^ mice in our facility. Both male and female mice were used in all experiments as no difference in disease pathogenesis between sexes was observed in our previous work (55).

### Corneal HSV-1 infection, clinical scoring, and corneal sensitivity.

Male and female 8-to-10-week-old mice were used in our experiments. Corneal infections were performed following anesthesia using ketamine/xylazine intraperitoneal (i.p.) injection. Corneas were scarified with a 25-gauge needle, and a volume of 2 × 10^6^ PFU of HSV-1 in 5 μl of Dulbecco’s modified Eagle medium (DMEM) was applied to each cornea. Mice were monitored daily for clinical symptoms using the following scoring system: 0, normal eye; 1, watery and swollen eye; 2, swollen eye and periocular lesion; 3, mild corneal haze; 4, moderate corneal opacity; 5, opaque cornea with corneal ulcer. A Luneau Cochet-Bonnet esthesiometer (catalog no. WO-7760; Western Ophthalmics, Lynnwood, WA, USA) was used to assess corneal sensitivity following infection. A 6.0-cm monofilament was touched to the surface of the cornea to elicit a blink response, and if no blink response was observed, the filament was shortened in 0.5-cm increments until a positive blink response was obtained. The absence of a blink response at the minimum filament length of 0.5 cm was recorded as a score of 0.

### Flow cytometry.

HSV-1-infected corneas were harvested and digested in 0.7 mg/ml Liberase (Roche, Indianapolis, IN, USA)–RPMI 1640 for 1 h in an incubator (37°C, 5% CO_2_). The corneas were homogenized using a syringe plunger on a 70-μm-pore-size mesh and washed with phosphate-buffered saline (PBS) to obtain single-cell suspensions. Cell counts were obtained using trypan blue and a Countess cell counter (Invitrogen, Carlsbad, CA, USA), and each sample was incubated with a 1:1,000 dilution of Live/Dead Fixable Aqua dead cell stain kit (Thermo Fisher Scientific)–PBS for 30 min. The samples were blocked with CD16/CD32 (eBioscience, San Diego, CA, USA) for 15 min in fluorescence-activated cell sorter (FACS) buffer (PBS plus 2% fetal bovine serum [FBS] plus 0.1% sodium azide). Single-cell suspensions of corneas, submandibular lymph nodes, and spleens were stained for cell surface markers using the following antibodies: from eBioscience, CD45 fluorescein isothiocyanate (FITC) (30-F11), CD3 allophycocyanin (APC)-eFluor 780 (17A2), CD11b phycoerythrin (PE)-Cy7 (M1/70), CD11c PE (N418), Ly6C peridinin chlorophyll protein (PerCP)-Cy5.5 (HK1.4), CD4 PE (GK1.5), CD3e PE-Cy7 (145-2C11), and CD62L APC (MEL-14); from BioLegend, Ly6G Brilliant Violet 421 (1A8), CD8a Brilliant Violet 421 (53-6.7), and BTLA PE (8F4); from BD Bioscience, CD4 APC-Cy7 (GK1.5), γδ T cell receptor (TCR) BV421 (GL3), and CD44 PE-Cy7 (IM7).

To detect cytokine expression in T cells, single-cell suspensions of corneas and LNs were activated for 4 h with 20 ng/ml phorbol 12-myristate 13-acetate 40 (PMA) and 1 mg/ml ionomycin in the presence of 2 mg/ml brefeldin A (Sigma-Aldrich, St. Louis, MO, USA). The cells were stained for surface markers following Live/Dead staining and were permeabilized and fixed using a Foxp3 intracellular staining kit (eBioscience). IL-17A Alexa Fluor 488 (ebio17B7) and IFN-γ APC (XMG1.2) were used for cytokine staining. The stained cells were acquired on a FACSCanto II analyzer (BD Biosciences, San Jose, CA, USA) and analyzed using FlowJo 10.1 software (Ashland, OR, USA).

### Anti-BTLA antibody treatment.

Corneal infections were performed on CD160^−/−^ LIGHT^−/−^ mice, and 200 μg of anti-BTLA MAb PJ196 (Bio X Cell, West Lebanon, NH, USA) or mouse IgG1 isotype control (Bio X Cell) was given i.p. every 48 h starting at −1 dpi and continuing to 11 dpi.

### Statistics.

All statistics were calculated using GraphPad Prism 7.0 software. For the clinical scoring and corneal sensitivity determinations, two-way analysis of variance (ANOVA) with Holm-Sidak’s multiple-comparison test was conducted. Viral titers, numbers of infiltrating immune cells, frequencies of effector CD4^+^ T cells, and frequencies of IFN-γ-producing CD4^+^ T cells were analyzed with one-way ANOVA and Holm-Sidak’s multiple-comparison test. The two-tailed *t* test with Holm-Sidak’s multiple-comparison was used for analysis of data collected from the anti-BTLA MAb treatment experiment.
